# Spatio-Temporal Epidemiology of Human West Nile Virus Disease in South Dakota

**DOI:** 10.3390/ijerph10115584

**Published:** 2013-10-29

**Authors:** Michael C. Wimberly, Paolla Giacomo, Lon Kightlinger, Michael B. Hildreth

**Affiliations:** 1 Geospatial Sciences Center of Excellence, South Dakota State University, Brookings, SD 57007, USA; E-Mail: paolla.giacomo@sdstate.edu; 2 South Dakota Department of Health, Pierre, SD 57501, USA; E-Mail: lon.kightlinger@state.sd.us; 3 Departments of Biology & Microbiology and Veterinary & Biomedical Sciences, South Dakota State University, Brookings, SD 57007, USA; E-Mail: michael.hildreth@sdstate.edu

**Keywords:** West Nile virus, disease map, seasonality, early detection, climate, physiography

## Abstract

Despite a cold temperate climate and low human population density, the Northern Great Plains has become a persistent hot spot for human West Nile virus (WNV) disease in North America. Understanding the spatial and temporal patterns of WNV can provide insights into the epidemiological and ecological factors that influence disease emergence and persistence. We analyzed the 1,962 cases of human WNV disease that occurred in South Dakota from 2002–2012 to identify the geographic distribution, seasonal cycles, and interannual variability of disease risk. The geographic and seasonal patterns of WNV have changed since the invasion and initial epidemic in 2002–2003, with cases shifting toward the eastern portion of South Dakota and occurring earlier in the transmission season in more recent years. WNV cases were temporally autocorrelated at lags of up to six weeks and early season cumulative case numbers were correlated with seasonal totals, indicating the possibility of using these data for short-term early detection of outbreaks. Epidemiological data are likely to be most effective for early warning of WNV virus outbreaks if they are integrated with entomological surveillance and environmental monitoring to leverage the strengths and minimize the weaknesses of each information source.

## 1. Introduction

Emerging infectious diseases, including those that have appeared for the first time, rapidly increased in incidence, or expanded into new geographic areas, are a significant concern in the fields of human and veterinary medicine as well as wildlife conservation [1,2]. Disease emergence is often connected with the development of human societies and their interactions with the environment, including the proliferation of transportation networks that facilitate pathogen spread, land use and climate change that affect habitats for arthropod vectors, and animal hosts, and population movements that increase human and domesticated animal contact with wildlife and their pathogens. The successful invasion of North America by West Nile virus (WNV) is a well-known example of infectious disease emergence that was initially caused by long-distance pathogen transport and facilitated by a diversity of suitable environments for transmission [3]. WNV is an arbovirus that is maintained in an enzootic cycle with mosquitoes as the primary vectors and birds as the primary reservoir hosts. Humans and horses are incidental hosts; both species can acquire the virus from infected mosquitoes but do not transmit the virus to uninfected mosquitoes and are not necessary for the maintenance of the pathogen.

WNV is indigenous to Africa, Asia, Europe, and Australia and was first identified in North America in the New York City metropolitan area during the summer of 1999 [3]. In 2002 widespread WNV cases occurred in the Midwest and south-central states, and the first WNV cases were reported in South Dakota. The epidemic wave moved further west in 2003, with extremely high incidence rates throughout the Great Plains and cases occurring as far west as California. From 2004 onward, WNV has been endemic across much of the western hemisphere with sporadic, localized outbreaks of human disease in a variety of geographic locations [4]. Human WNV disease has had a significant public health impact in the United States. A total of 37,088 WNV cases have been reported to the Centers for Disease Control through 2012, including 16,196 cases of severe neuroinvasive disease and 1,549 deaths. The widespread resurgence of human WNV disease in 2012 following several years of relatively low incidence has highlighted the continued public health hazard posed by WNV and emphasized the need for more accurate predictions of when and where WNV outbreaks will occur.

Disease mapping and exploratory spatial analysis of epidemiological data can help to identify the geographic locations of populations at risk and facilitate the development of hypotheses about the epidemiological and ecological processes that drive these patterns [5,6]. Time-series analysis can similarly elucidate seasonal cycles, longer-term trends, and deviations from these expected patterns that indicate the times when WNV risk is highest and suggest new hypotheses about the drivers of temporal variability at different scales [7,8]. In particular, early detection of anomalously high human case numbers, particularly during the early part of the WNV season, may serve as a harbinger of developing outbreaks [9]. There are a number of limitations to inferring spatial and temporal patterns of disease risk from human disease cases, particularly in rural areas with low human densities and in situations where time lags in disease case reporting and confirmation delay the availability of these data. However, human disease surveillance can still provide valuable information that complements other sources of information from mosquito surveillance [10], dead bird surveillance [11], and ecological forecasting based on environmental monitoring data [12].

Since its arrival in 2002, WNV has had a significant public health impact in South Dakota and neighboring Great Plains states. From 1999–2008 South Dakota had the highest average incidence of WNV neuroinvasive disease in the United States [13]. During 2012, a year in which WNV reemerged at a national level, South Dakota once again had the highest incidence rates of both neuroinvasive WNV disease (7.5/100,000), total WNV disease (24.6/100,000 including WNV fever in addition to WNV neuroinvasive disease), and viremic blood donors (5.1/100,000). The high and persistent incidence of WNV in South Dakota and the surrounding region has been hypothesized to reflect the geographic distribution of *Culex tarsalis*, a highly ornithophilic and particularly efficient amplifying vector of WNV that can also serve as a bridge vector to humans [14]. Previous WNV research in South Dakota found that *Culex tarsalis* abundance was lowest in urban areas and highest in grass-dominated rural habitats [15], and the incidence of human WNV disease was similarly highest in rural areas with poorly drained soils [16]. Seasonal and interannual variability in *Culex tarsalis* abundance in South Dakota exhibited much stronger lagged relationships with temperature than with precipitation [15,17], and a high number of accumulated degree days during the spring and early summer was found to be an indicator of the risk of human WNV disease during the subsequent main transmission season in mid to late summer [18].

The overarching goal of our study was to extend this previous work by characterizing the spatio-temporal epidemiology of WNV disease in South Dakota from 2002–2012. The underlying rationale was the expectation that identifying spatial and temporal patterns of human disease occurrence will provide a clearer understanding of when and where WNV risk is the highest and whether these patterns have changed since the emergence of WNV in South Dakota. We addressed the following specific questions: (1) Where in South Dakota is WNV risk highest and has this geographic pattern changed over time? (2) During what part of the year is WNV risk highest and has this seasonal pattern changed over time? (3) What are the temporal patterns (deviations from expected seasonal cycles and long-term trends) of WNV anomalies and can they be used to predict future WNV risk? (4) Do WNV epidemics arise simultaneously throughout the state or do cases increase earlier in some geographic locations than in others?

## 2. Methods

**Figure 1:**
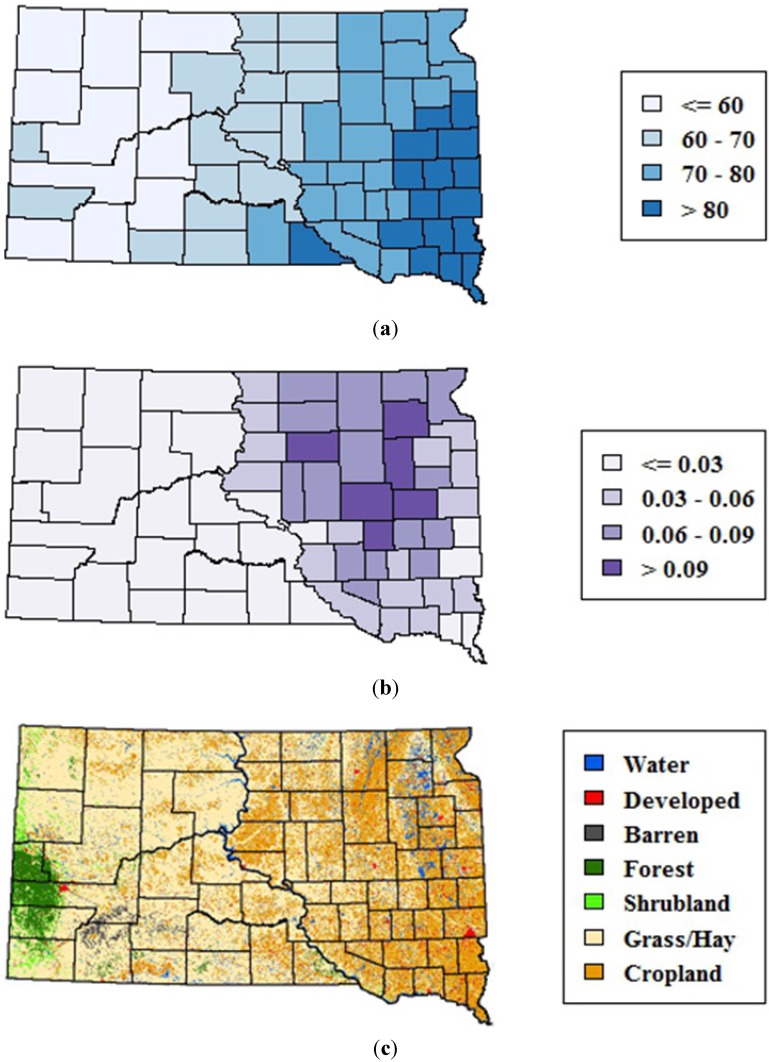
Environmental variability across South Dakota. (**a**) 1982–2011 mean monthly precipitation (mm) from May–September summarized at the county level (**b**) Emergent wetlands from the National Wetlands Inventory summarized as proportion of total county area, and (**c**) 2006 map of major land cover classes from the National Land Cover Database.

**Figure 2:**
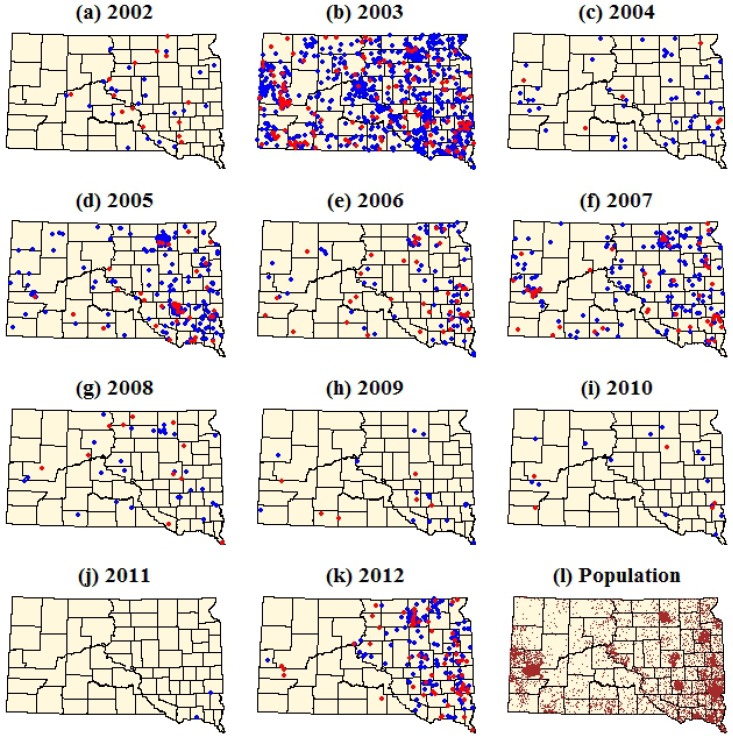
Dot density maps of WNV cases in South Dakota from 2002–2012. (**a–k**) Each dot represents a single case within the ZIP code tabulation area (ZCTA) of the patient’s residence. Red dots represent WNV neuroinvasive disease and blue dots represent WNV fever. (**l**) Brown dots represent total population with one dot per 100 people. Dots were assigned random locations within the ZCTAs for the purpose of visualization.

The study area encompassed the state of South Dakota, which is located in the northern Great Plains of the United States, a region historically dominated by prairie vegetation. Environmental variability across the state is strongly affected by precipitation, which generally decreases along an east-to-west gradient (Figure 1(a)). This precipitation gradient is reflected in the distribution of wetlands, which are highest in the eastern portions of the state (Figure 1(b)). Land cover patterns also vary along this gradient, ranging from a mixture of row crops, pasture, hay, and grasslands in eastern South Dakota to predominantly rangelands in the drier western region (Figure 2(c)). Forests are mostly restricted to the Black Hills on the far western edge of the state.

Epidemiological data on human WNV disease cases were collected by the South Dakota Department of Health. Each case was identified by disease type (WNV fever or neuroinvasive disease), referenced temporally by the reported date of disease onset, and referenced spatially by county, ZIP code, and city of residence. Each case was georeferenced using the 2010 map of ZIP code tabulation areas (ZCTAs) from the U.S. Census Bureau. A total of 23 cases (1.1% of the total number) were missing ZIP codes and were assigned to a ZCTA based on the county and city of residence. In addition, 60 cases (2.8% of the total number) had ZIP codes that were not present in the 2010 ZCTA map and were assigned to a 2010 ZCTA by referencing the 2000 ZCTA map or based on the county and city of residence. The 2010 population data for each ZCTA were also obtained from the U.S. Census Bureau.

ZIP codes were developed by the U.S. Postal Service to classify street segments, address ranges and delivery points for mail delivery. ZCTAs were created by the U.S. Census Bureau as spatial units to provide approximate mapping of ZIP code boundaries using aggregated Census blocks. However, it is difficult to map ZIP code boundaries precisely, and ZIP codes are frequently split, discontinued, added, or expanded [19]. As a result, some ZIP codes may not have a corresponding ZCTA and some addresses may actually be located in different ZCTAs relative to their ZIP code. Despite these limitations, ZIP code level data has the advantages of greater spatial precision than coarser county-level summaries and higher rates of geocoding success than street address-level geocoding [20,21]. The 2010 ZCTAs in South Dakota had a mean area of 519 km^2^ and a mean population of 2,115. The mean population density at the ZCTA level was 34 per km^2^, but individual ZCTAs ranged from 0.05 to 4,628 per km^2^ reflecting the variety of settlement patterns from dense cities to rural areas.

Spatial patterns of WNV cases were displayed as dot density maps by selecting a random location for each case within its assigned ZCTA. Spatial patterns of WNV risk were mapped using annual incidence rates and were spatially smoothed by incorporating information from surrounding areas to reduce variability caused by small population sizes at the ZCTA level. For this initial assessment of WNV risk, smoothing was carried out using a simple population-weighted mean smoothing approach as suggested by Waller and Gotway [6]. To generate the smoothed maps, a neighborhood mean of WNV cases was calculated for each ZCTA as the mean number of cases from the focal ZCTA and all ZCTAs with centroids falling within a 75 mile radius of the focal ZCTA. A neighborhood mean of the 2010 population was calculated for each ZCTA using the same method. The annual incidence rate, normalized to a population of 100,000, was calculated for each ZCTA using these smoothed values. To explore changes in the spatial pattern of WNV risk over time, smoothed maps were generated for each of three time periods: 2002–2003, 2004–2007, and 2008–2012. These time periods were subjectively selected to capture three distinctive epochs of WNV transmission in South Dakota: the initial invasion and first large epidemic (2002–2003), a subsequent period of relatively high WNV incidence (2004–2007), and a period of relatively low WNV incidence followed by the resurgence of human WNV cases in 2012 (2008–2012). Separate sets of maps were generated for the total incidence of human WNV disease (including both WNV fever and neuroinvasive disease) and for only WNV neuroinvasive disease.

Seasonal patterns of total human WNV cases were summarized based on a standardized 18-week season in which all cases prior to 1 June were included in week 1; the months of June (weeks 2–5), July (weeks 6–9), August (weeks 10–13), and September (weeks 14–17) were each divided into four weeks; and all cases after September 30th were included in week 18. Weekly cumulative totals of human WNV cases were graphed for each year and for the three time periods used in the mapping (2002–2003, 2004–2007, and 2008–2012). Two-sample Kolmogorov-Smirnov tests were used to test for differences in the seasonal distribution of WNV cases among these three time periods. These tests were carried out using all human WNV cases (including both WNV fever and neuroinvasive disease), only the WNV fever cases, and only the WNV neuroinvasive disease cases.

The 18 weeks of WNV data for each year were concatenated to generate a continuous time series of WNV cases from 2002–2012. This log-transformed time series was then decomposed into seasonal, trend, and remainder components with the seasonal and trend decomposition using loess (STL) procedure [22]. In this approach, the seasonal component was calculated as the mean number of cases in each week across all years and the long-term trend was estimated using a smoothed locally-weighted regression. The remainder component reflected anomalies of WNV cases relative to these longer term patterns, and was computed by subtracting the seasonal and long-term trends from the raw data. Temporal autocorrelation was assessed by computing autocorrelation functions at time lags ranging from 1 to 18 weeks for both the raw data and the remainders from the STL decomposition.

WNV cases were also analyzed to determine whether the numbers of cases occurring during the early part of the WNV season (June and July) were predictive of the total number of WNV cases occurring throughout the entire season. For this analysis, we summarized the cumulative total number of WNV cases occurring in each year from week 2 (the first week in June) through week 9 (the last week in July). We computed the Spearman rank correlation between each of these cumulative variables and the total number of cases occurring for 2004–2012 (*N* = 9). We examined the changes in these correlations across the different weeks to determine the point during the season when WNV cases provided a reliable indicator of the total magnitude of WNV cases throughout the season.

We conducted an analysis of space-time interactions during major WNV outbreak years to determine whether there were significant changes in the geographic locations of WNV cases throughout the season. To have sufficient data for the analysis, we focused on major outbreak years during which more than 200 WNV cases occurred in South Dakota (2003, 2005, 2007, and 2012). For each of these years, we used a generalized additive model (GAM) to model the day of year (DOY) of each case as the dependent variable using a smoothed functions of the spatial coordinates (eastings and northings) of the ZCTA centroids as independent variables. Statistically significant relationships between DOY and one or both of the independent variables indicated that different geographic locations had different mean dates of WNV onset. For years where there was significant spatial variability in mean DOY, the models were applied across the entire state to produce a continuous map of the mean DOY of WNV cases for each ZCTA.

## 3. Results

The dot density maps emphasized the strong spatial and temporal heterogeneity of human WNV cases within South Dakota (Figure 2, Table 1). During 2002, the first year of WNV occurrence in South Dakota, a relatively small number of WNV cases were concentrated in the eastern portion of the state. In 2003 there was a major increase in both WNV fever and neuroinvasive disease, with several significant clusters occurring in the central and western parts of South Dakota. WNV cases declined in 2004, followed by a three year period (2005–2007) of increased WNV cases. Annual numbers of WNV cases declined steadily over the next four years (2008–2011), reaching their lowest levels in 2011 when only two cases of WNV fever were reported. In 2012, there was a significant resurgence of WNV cases in South Dakota, with a stronger concentration of these cases in the eastern part of the state relative to previous years. Annual, log-transformed fever case counts and neuroinvasive case counts were strongly correlated at the state level (*r* = 0.96, *p* < 0.001).

**Table 1:** Annual cases of human West Nile virus disease in South Dakota.

Year	Fever	Neuroinvasive	Total Cases	Deaths
2002	23	14	37	0
2003	868	171	1,039	13
2004	45	6	51	1
2005	194	35	229	2
2006	75	38	113	3
2007	160	48	208	6
2008	28	11	39	0
2009	15	6	21	0
2010	16	4	20	0
2011	2	0	2	0
2012	141	62	203	3

**Figure 3:**
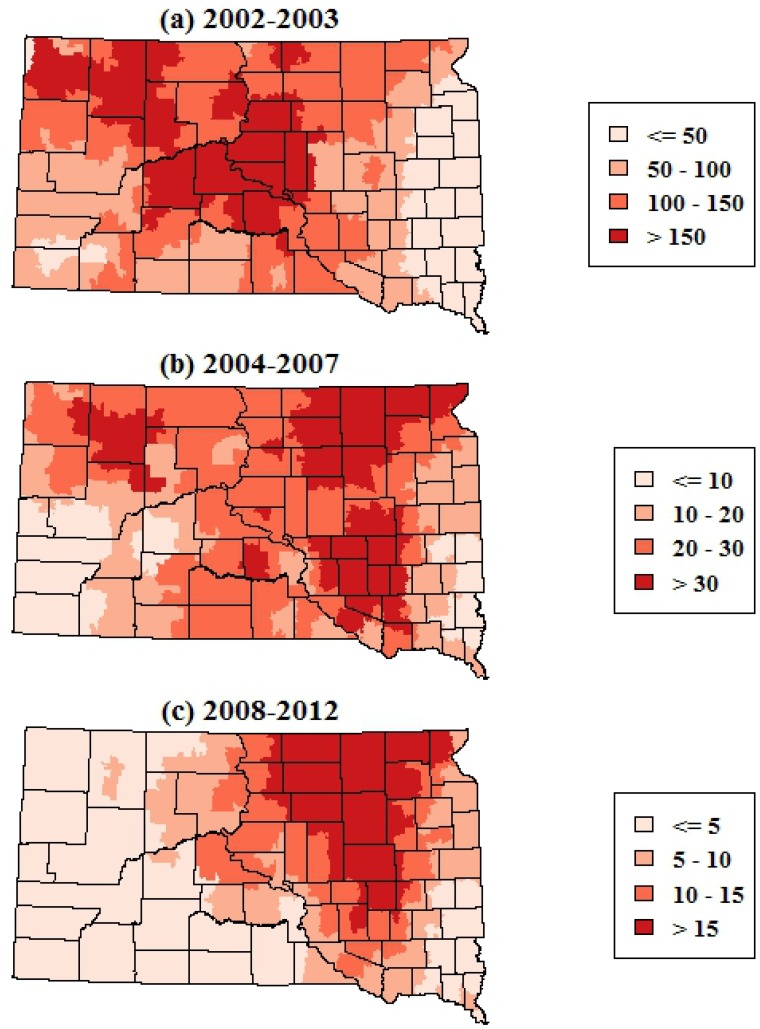
Spatially smoothed incidence rates of all human WNV disease (including both fever and neuroinvasive disease) in South Dakota for three time periods. Rates are the annual number of cases per 100,000 population.

**Figure 4:**
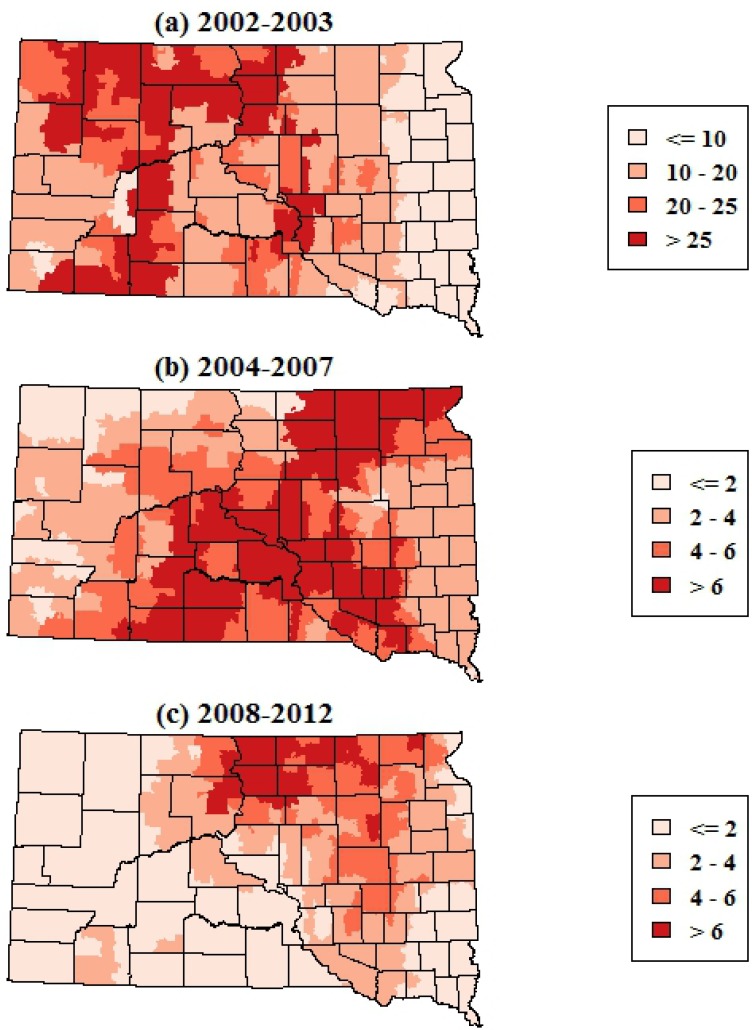
Spatially smoothed incidence rates of human WNV neuroinvasive disease in South Dakota for three time periods. Rates are the annual number of cases per 100,000 population.

The smoothed maps of WNV incidence further highlighted changes in the geographic distribution of WNV risk over time. The maps of total WNV incidence showed a shift in the geographic patterns of risk from central and western portions of South Dakota in 2002–2003 to the eastern portion of the state in 2004–2007 and 2008–2012 (Figure 3). The smoothed maps of WNV neuroinvasive disease incidence showed more spatial variability at finer scales, reflecting the smaller numbers of neuroinvasive cases (Figure 4). The smoothed estimates of total WNV incidence and neuroinvasive WNV incidence were strongly correlated in 2002–2003 (*r* = 0.75, *p* < 0.001), exhibited a weaker correlation in 2004–2007 (*r* = 0.66, *p* < 0.001), and had the highest correlation in 2008–2012 (*r* = 0.86, *p* < 0.001). As with the maps of total WNV cases, the incidence of neuroinvasive disease shifted from central and western South Dakota in 2002–2003 toward the eastern portion of the state in 2004–2007 and 2008–2012.

**Figure 5:**
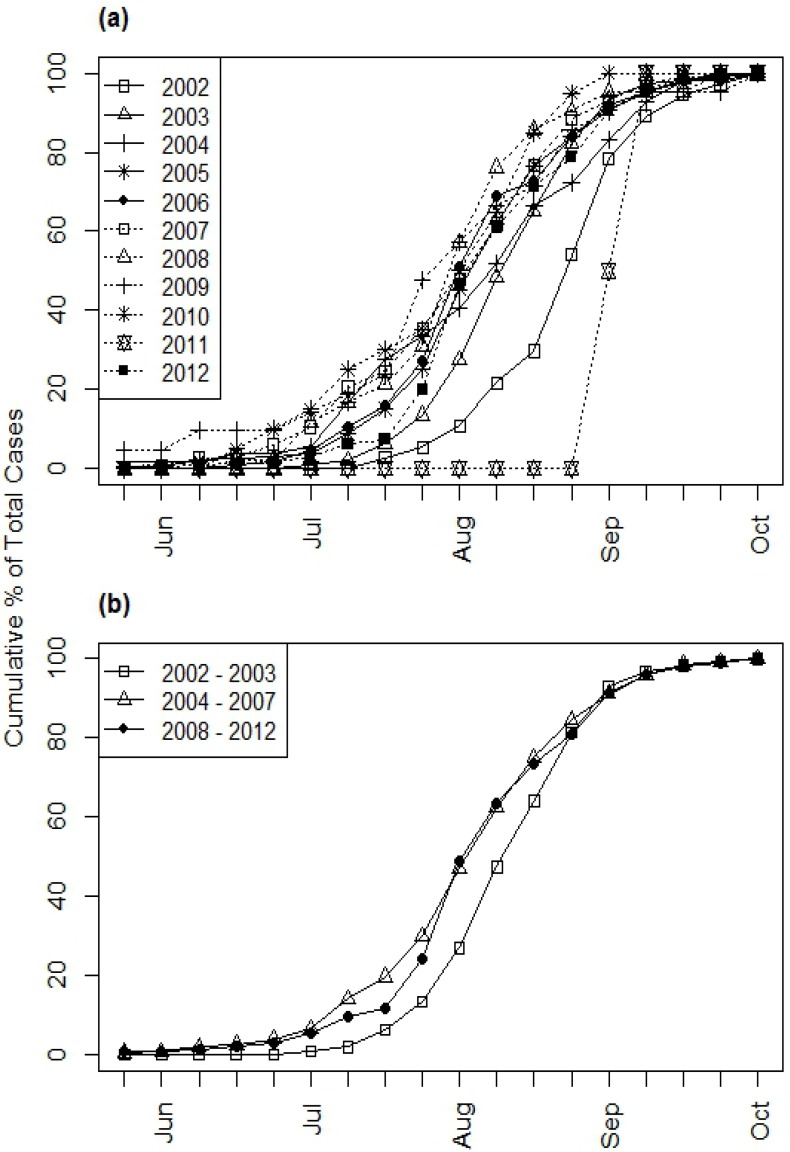
Weekly cumulative distributions of illness onsets of all human WNV cases (including both fever and neuroinvasive disease) in South Dakota from 2002–2012. (**a**) Annual curves, (**b**) Combined curves for three time periods.

Seasonal patterns of WNV cases varied from year to year (Figure 5(a)). For example, 5% of the cumulative WNV cases occurred before the end of July in 2002, whereas 14% of the cumulative WNV occurred before the end of July in 2003 and 20–48% of cases occurred before the end of July in all of the subsequent years except 2011. 2011 stood out as an anomalous year in which only two WNV cases occurred, both in September. The combined seasonal distributions for the 2002–2003, 2004–2007, and 2008–2012 time periods also illustrated a shift toward earlier occurrence of WNV cases after 2003 (Figure 5(b)). During all three time periods, the majority of WNV cases occurred during the last week of July and the first three weeks of August (58% in 2002–2003, 55% in 2004–2007, and 62% in 2008–2012). However, only 6% of cases occurred before the last week of July in 2002–2003 compared to 20% in 2004–2007 and 12% in 2008–2012. In contrast, 36% of cases occurred after the third week of August in 2002–2003 compared to 25% in 2004–2007 and 26% in 2008–2012. The Kolmogorov-Smirnov tests found statistically significant differences in WNV seasonality in 2002–2003 compared to 2004–2007 (D = 0.2016, *p* < 0.001) and in 2002–2003 compared to 2008–2012 (D = 0.2195, *p* < 0.001), but the difference was not statistically significant in 2004–2007 compared to 2008–2012 (D = 0.08, *p* = 0.12). Similar results were obtained when only the same tests were performed using only the WNV fever cases and only the WNV neuroinvasive disease cases.

**Figure 6:**
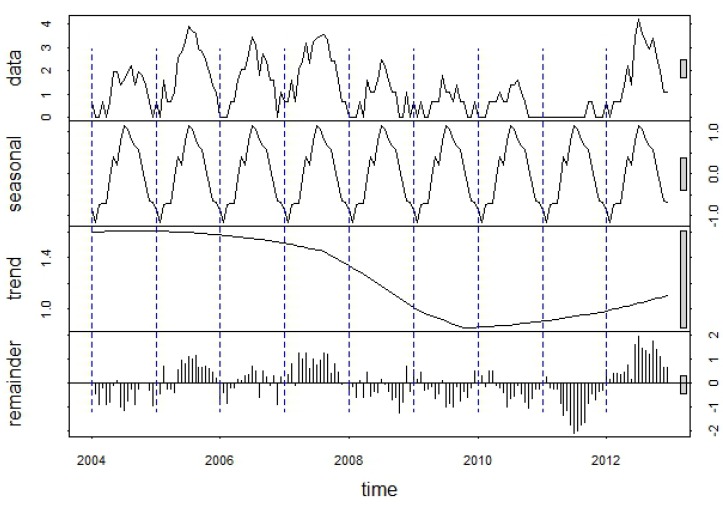
Decomposition of the log-transformed 2004–2012 WNV time series into seasonal, trend, and remainder components using the STL procedure. Each “year” in the time series consists of an 18-week WNV season extending from the last week in May through the first week in October.

**Figure 7:**
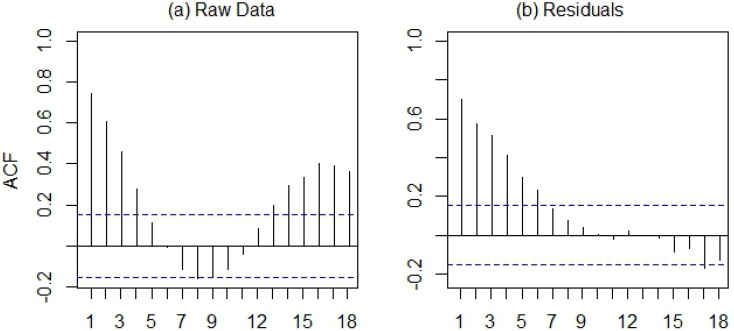
Temporal autocorrelation function for (**a**) the raw, log-transformed time series of WNV cases and (**b**) the remainders from the time series decomposition. Blue lines represent critical values for statistical significance of the autocorrelation statistic at an alpha-level of 0.05.

These patterns were reflected in the seasonal component of the WNV time series decomposition, which showed a strong seasonal peak between the last week of July and the third week of August (Figure 6). The smoothed trend component illustrated longer-term variation in human WNV cases, with highest case numbers from 2003 through 2006, declining case numbers after 2007, and an increase driven by the 2012 outbreak. The raw, log-transformed WNV case data exhibited positive temporal autocorrelation at lags from 1 to 4 weeks and from 13 to 18 weeks (Figure 7(a)), reflecting the previously-described seasonal pattern of WNV. After removing the seasonal patterns and long-term trend from the data, the remainder exhibited positive temporal autocorrelation at lags from 1 to 6 weeks (Figure 7(b)). This pattern suggested that anomalously high numbers of WNV cases can serve as indicators of continued high WNV risk up to 6 weeks in the future. Cumulative case counts through the first and second weeks of June were not significantly correlated with annual case counts at an alpha-level of 0.05 (Table 2). However, cumulative case counts from the third week of June were significantly correlated with annual case counts, and the magnitude of this correlation generally increased as more cases were accumulated later into the season.

**Table 2:** Spearman rank correlations between cumulative case numbers through July and August and total annual case numbers from 2004–2012 (*N* = 9).

Month	Week	Rho	*p*-value
June	1	0.603	0.086
	2	0.656	0.055
	3	0.783	0.013
	4	0.801	0.010
July	1	0.882	0.002
	2	0.917	0.001
	3	0.867	0.005
	4	0.933	<0.001

**Table 3:** Results of generalized additive models predicting the day of year of WNV case onset as a function of the eastings and northings of their respective ZCTAs.

Year	*N*	*p*-value		Adjusted R^2^
		Easting	Northing	
2003	1,041	<0.001	0.0112	0.0617
2005	229	0.0052	0.2409	0.0386
2007	208	0.0005	0.3562	0.0746
2012	202	0.4100	0.2990	0.0267

**Figure 8:**
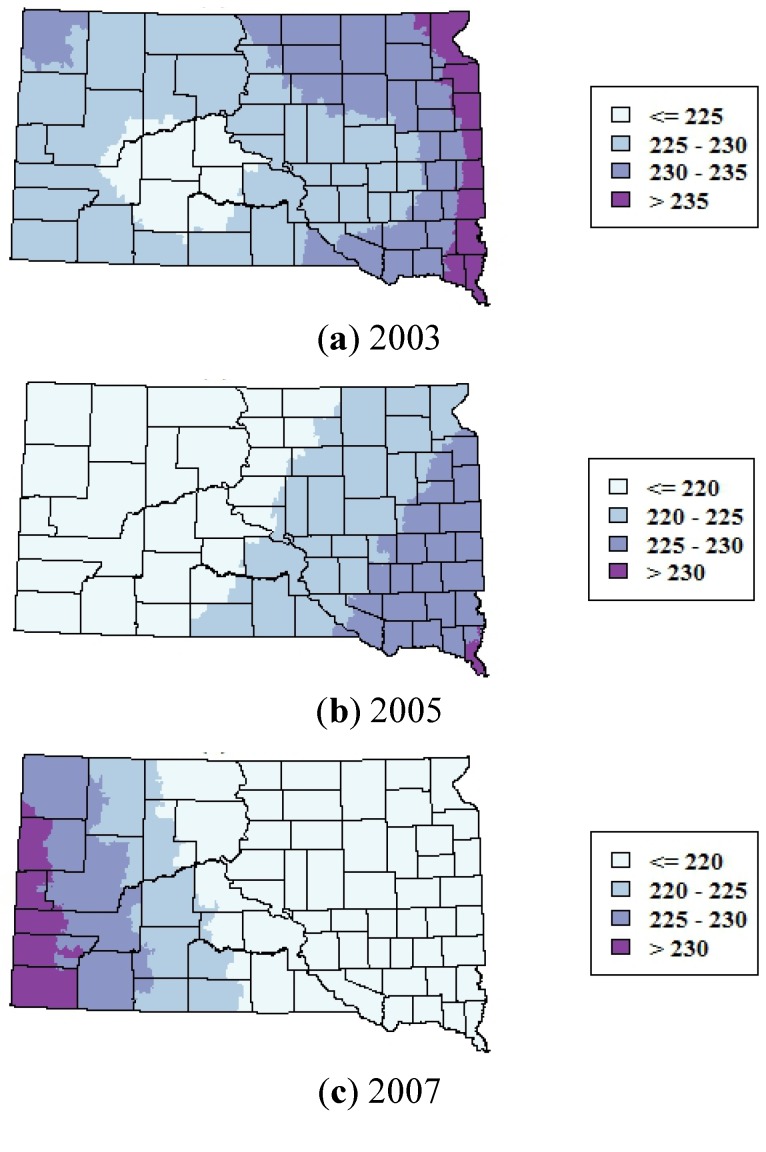
Smoothed maps of the mean day of year of WNV cases for 2003, 2005, and 2007. Maps were generated by using generalized additive models to predict the DOY as a smoothed function of the spatial coordinates of the ZCTAs.

There was statistically significant variability in the onset date of WNV cases in both the north-south and east-west directions in 2003, and in the east-west direction in 2005 and 2007 (Table 3). There were no statistically significant spatial trends in the onset date of WNV cases in 2012. In 2003 and 2005, cases tended to occur earlier in the western part of the state and later in the season in the eastern part of the state (Figure 8). The reverse pattern occurred in 2007; cases tended to occur earlier in the eastern part of the state and later in the western part of the state. The adjusted R^2^ values of the GAMs were all low (<0.08), indicating that these space-time patterns were relatively weak.

## 4. Discussion

There has been a shift in the spatio-temporal niche of human WNV risk in South Dakota from the initial invasion and first major epidemic of WNV in 2003–2003 to the subsequent years of endemic WNV from 2004-onward. In 2002–2003, human WNV cases were broadly distributed across the state and in particular were higher in the central and western portions. Starting in 2004, cases were more concentrated in the eastern portion of the state, and this eastward shift became even more pronounced during the 2008–2012 period. In addition, there were significantly fewer cases in the early part of the WNV season (before the last week of July) and more cases in the later part of the WNV season (after the third week of August) in 2002–2003 compared to 2004 and later years. These changes suggest that the spatial and temporal patterns observed during the large 2003 epidemic have become less relevant to current ecological and epidemiological processes that drive WNV risk, and that disease maps and other inferences about human WNV risk should be based on epidemiological data from 2004 onward.

Climatic variability is one possible explanation for the observed changes. A geographic analysis of the 2003 WNV epidemic in the northern Great Plains highlighted strong clustering of WNV in the Dakotas, Nebraska, and eastern Montana and Wyoming [14]. This cluster occupied a climatic niche characterized by higher temperatures and intermediate precipitation, and was hypothesized to reflect the geographic distribution of *Culex tarsalis*, a particularly efficient WNV vector. Subsequent research showed that interannual variability in temperature and moisture during the spring and early summer was associated with spatial and temporal variability in WNV incidence across the region from 2004–2011 [18]. Thus, anomalies in the regional patterns of temperature and precipitation in 2002 and 2003 relative to later years may explain some of the differences in geographic pattern and seasonality. Alternately, changes in geographic and seasonal patterns of WNV cases after 2003 may reflect other ecological and epidemiological factors, such as the development of immunity in both human and avian populations and consequent impacts on virus amplification in avian communities and viral transmission to humans. In addition, significant and persistent declines in the abundances of multiple bird species resulting from WNV-caused mortality have been documented across the United States [23,24]. Resulting changes in bird community composition across South Dakota have the potential to influence the spatial pattern and timing of virus amplification and risk to humans, but have been unexplored to date and merit further investigation.

From 2004 onward, and particularly in 2008–2012, one of the regions of highest WNV risk within South Dakota was concentrated in eastern South Dakota within the James River Valley, which lies between the uplands of the Missouri Couteau to the West and the Prairie Couteau to the East. This broad lowland was created by the James Lobe of the Laurentide ice sheet and occupies approximately 49,000 km^2^. It is characterized by a high density of seasonal and permanent wetlands and a landscape mosaic of row crops and grasslands (Figure 1), and is currently being impacted by land use changes that are resulting in widespread wetland drainage and conversion of grasslands to row crop agriculture [25,26]. Previous studies have highlighted spatial clustering and climatic associations of human WNV cases at national and regional levels [14,27] and finer-scale associations of human WNV disease with land cover, land use, soils, and hydrology at the landscape level [16,28,29]. The geographic patterns highlighted in this study further suggest that broad physiographic features such as the James River Valley can influence WNV risk to humans through their characteristic hydrological processes, land use patterns, and consequent effects on habitat for vector and host populations. More research is needed to better elucidate the proximal determinants of the geographic distribution WNV risk at the state level and to apply this knowledge to improve strategies for WNV prevention and control.

Early detection systems are widely used to identify the onset of mosquito-borne disease outbreaks by tracking human disease cases. Forecasting techniques range from relatively simple methods based on thresholds determined from historical data [30], to more sophisticated statistical methods for time series forecasting [31]. Our results suggest that anomalously high numbers of WNV cases, relative to seasonal patterns and longer-term trends, provide an indicator of continued high numbers of WNV cases up to 6 weeks in the future. Alternately, the cumulative number of WNV cases through the end of June could be used to make a seasonal forecast of the total number of expected cases during the peak of the WNV season in July and August. Although statistically significant spatio-temporal patterns were detected in the major outbreak years, the relationships were relatively weak and variable from year to year. As a result, there currently appears to be a relatively low potential for using human case data to predict the precise locations of WNV risk within South Dakota at specific times during the WNV season.

Two major limitations to the use of human surveillance data for forecasting disease outbreaks are data quality and data availability. For human WNV disease in the United States, all cases are assessed based on a consistent definition established by the Centers for Disease Control and are subject to laboratory confirmation. However, an unknown degree of spatial uncertainty is introduced because some patients may contract WNV at a location away their residence. In addition, the delay introduced as a result of the time lags between amplification of the virus in mosquito and bird populations, transmission to humans, onset of symptoms, medical diagnosis, and case confirmation mean that human cases are a lagging indicator of WNV risk. As result, WNV amplification will have already occurred and mosquito numbers and infection rates will likely already be high by the time WNV is detected in the human population. In a state like South Dakota that experiences human WNV cases every year, detection of anomalies in early season case data does have some potential to predict the magnitude of WNV in human populations during the peak transmission season. In particular, our results suggest that human surveillance data may be able to indicate the onset of a significant WNV outbreak with several weeks of lead time. This type of alert would not provide enough time to take action to prevent virus amplification, but it might still allow public health officials to take steps such as expanding adult mosquito control and issuing preventive warnings before the period of peak transmission to humans.

One promising approach toward leveraging the strengths and minimizing the limitation of human case surveillance for disease outbreak detection is to use blended approaches that integrate multiple streams of surveillance data. For example, a study of WNV in Colorado combined environmental data, entomological data, and human WNV case data to develop an improved map of disease risk in the Northern Colorado Front Range [32]. Research on malaria early warning in the highlands of Ethiopia has found that forecasting models that incorporate both remotely-sensed climatic variables and lagged indicators from human case surveillance are more effective at predicting epidemics than models based on either environmental data alone or human cases alone [31,33]. The California Mosquito-Borne Virus Surveillance and Response Plan incorporates multiple sources of information, including meteorological data, vector abundance, vector infection rates, sentinel chicken seroconversion, dead bird reporting and testing, and human surveillance into a single index [34,35]. This multifactor index was found to be a better early warning indicator for the onset of human WNV disease than predictions based on single sources of information [35]. Previous research in South Dakota has found influences of climatic variability on the abundance of vector mosquitoes [15,17] and human disease cases [18] and we suggest that combining these types of environmental indicators with mosquito and human case surveillance data offers a strong potential for improving our ability to predict the spatial and temporal dimensions of WNV in South Dakota as well as in other regions where WNV is endemic.

## 5. Conclusions

Human WNV disease exhibits strong patterns of geographic variability, seasonal cycles, and interannual fluctuations in South Dakota. The geographic and seasonal patterns of WNV have changed since the invasion and initial epidemic in 2002–2003, with cases shifting toward the eastern portion of South Dakota and occurring earlier in the transmission season in more recent years. The underlying cause of these shifts is currently unknown, but the results suggest that more recent data on endemic WNV from 2004 onward should be used for risk mapping and assessment of seasonality for public health planning purposes. Temporal analyses indicate that there is a potential for forecasting upcoming large WNV outbreaks with lead times of up to several weeks. Although this time frame is likely insufficient for preventing virus amplification, it could still help reduce transmission to humans by providing time to ramp up emergency mosquito control and disseminate messages to the public to take personal protection measures. Ultimately, information from epidemiological surveillance should be integrated with environmental monitoring, entomological surveillance, and other sources of information to provide more effective predictions of the likelihood of future WNV outbreaks.
